# 2,3-Dimeth­oxy-10-oxostrychnidinium hydrogen oxalate dihydrate

**DOI:** 10.1107/S1600536813008623

**Published:** 2013-04-05

**Authors:** P. Krishnan, K. Gayathri, N. Sivakumar, G. Chakkaravarthi, G. Anbalagan

**Affiliations:** a Department of Physics, Presidency College, Chennai 600 005, India; b Department of Physics, CPCL Polytechnic College, Chennai 600 068, India

## Abstract

In the cation of the title salt, C_23_H_27_N_2_O_4_^+^·C_2_HO_4_^−^·2H_2_O, both fused pyrrolidine rings exhibit twisted conformations, while the piperidine rings adopt screw-boat and boat conformations. In the crystal, the three components are linked *via* O—H⋯O and N—H⋯O inter­actions, forming a tape along the *b* axis. The tapes are further linked by weak C—H⋯O hydrogen bonds. forming a three-dimensional network.

## Related literature

For related structures, see: Smith *et al.* (2005 ▶, 2006 ▶).
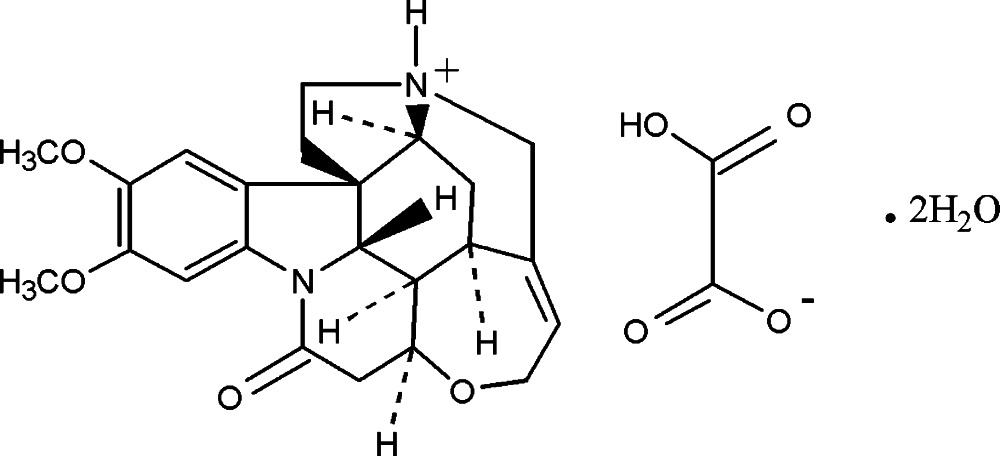


## Experimental

### 

#### Crystal data

C_23_H_27_N_2_O_4_^+^·C_2_HO_4_^−^·2H_2_O
*M* *_r_* = 520.53
Orthorhombic, 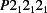

*a* = 7.6110 (2) Å
*b* = 10.7375 (3) Å
*c* = 29.4990 (7) Å
*V* = 2410.75 (11) Å^3^
*Z* = 4
Mo *K*α radiation
μ = 0.11 mm^−1^
*T* = 295 K
0.28 × 0.24 × 0.20 mm

#### Data collection

Bruker Kappa APEXII CCD diffractometer
Absorption correction: multi-scan (*SADABS*; Sheldrick, 1996 ▶) *T*_min_ = 0.970, *T*_max_ = 0.978
13171 measured reflections
5805 independent reflections
5363 reflections with *I* > 2σ(*I*)
*R*_int_ = 0.020

#### Refinement

*R*[*F* ^2^ > 2σ(*F* ^2^)] = 0.042
*wR*(*F* ^2^) = 0.116
*S* = 1.05
5805 reflections
354 parameters
7 restraints
H atoms treated by a mixture of independent and constrained refinement
Δρ_max_ = 0.33 e Å^−3^
Δρ_min_ = −0.32 e Å^−3^

### 

Data collection: *APEX2* (Bruker, 2003 ▶); cell refinement: *SAINT* (Bruker, 2003 ▶); data reduction: *SAINT*; program(s) used to solve structure: *SHELXS97* (Sheldrick, 2008 ▶); program(s) used to refine structure: *SHELXL97* (Sheldrick, 2008 ▶); molecular graphics: *PLATON* (Spek, 2009 ▶); software used to prepare material for publication: *SHELXL97*.

## Supplementary Material

Crystal structure: contains datablock(s) I, global. DOI: 10.1107/S1600536813008623/is5247sup1.cif

Structure factors: contains datablock(s) I. DOI: 10.1107/S1600536813008623/is5247Isup2.hkl

Additional supplementary materials:  crystallographic information; 3D view; checkCIF report

## Figures and Tables

**Table 1:** Hydrogen-bond geometry (Å, °)

*D*—H⋯*A*	*D*—H	H⋯*A*	*D*⋯*A*	*D*—H⋯*A*
N2—H2*B*⋯O8	0.88 (1)	1.87 (1)	2.712 (2)	159 (2)
O5—H5*A*⋯O7^i^	0.83 (1)	1.83 (1)	2.652 (2)	170 (4)
O9—H9*A*⋯O3^ii^	0.85 (1)	2.04 (2)	2.832 (3)	155 (5)
O9—H9*B*⋯O10	0.84 (1)	2.40 (3)	3.161 (4)	151 (5)
O10—H10*D*⋯O8	0.82 (1)	2.04 (1)	2.854 (3)	173 (5)
C7—H7⋯O9^iii^	0.98	2.50	3.456 (3)	166
C11—H11⋯O9^iv^	0.98	2.56	3.519 (3)	168
C17—H17*A*⋯O1^ii^	0.97	2.56	3.518 (2)	168
C21—H21*A*⋯O6^v^	0.97	2.43	3.188 (3)	135
C21—H21*B*⋯O5^vi^	0.97	2.59	3.293 (3)	130
C14—H14*B*⋯O1^vii^	0.97	2.49	3.291 (2)	139

Symmetry codes: (i) 
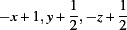
; (ii) 
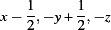
; (iii) 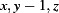; (iv) 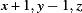; (v) 
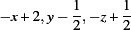
; (vi) 
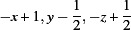
; (vii) 
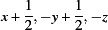
.

**Fig. 1.**
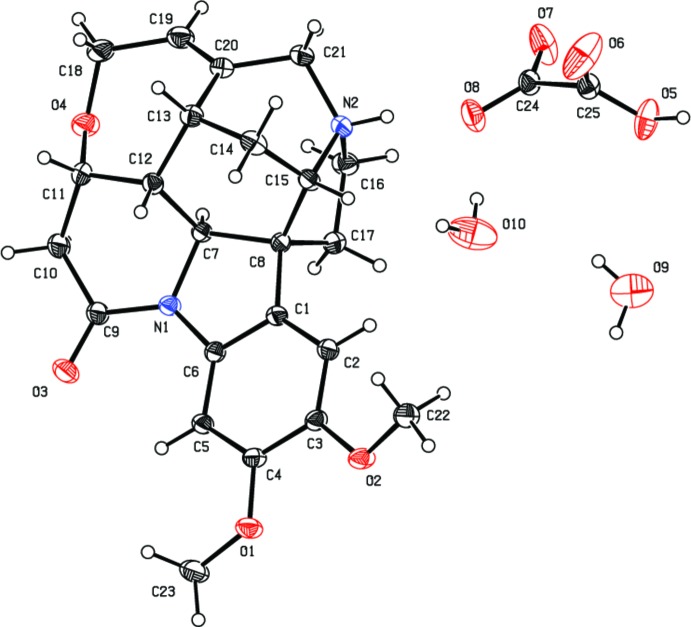
The molecular structure of the title compound, with atom labels and 30% probability displacement ellipsoids for non-H atoms.

**Fig. 2.**
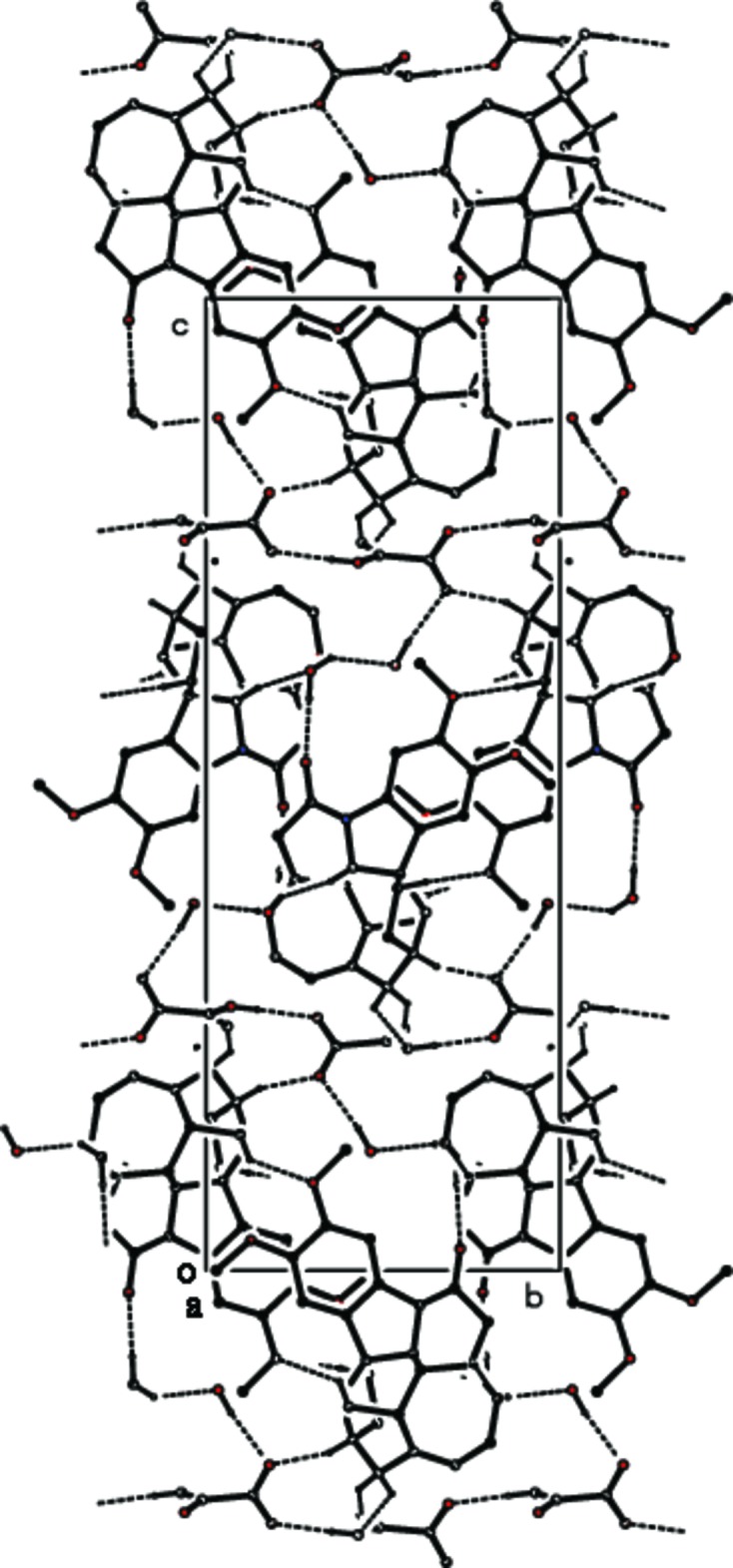
The packing of the title compound, viewed down the a axis. Hydrogen bonds are shown as dashed lines.

## supplementary crystallographic information

### Comment

The strychnos alkaloids strychnine and brucine have mostly been used to resolve enantiomorphic mixtures of chiral compounds, and the number of crystal structures of both salts and adducts of strychnine (Smith *et al.*, 2006).

The geometric parameters of the title compound (Fig. 1) agree well with reported similar structure (Smith *et al.*, 2005,2006). In the cation, both the pyrrolidine rings exhibit twisted conformations. The sum of bond angles around N1 [354.9 (2)°] and N2 [330.4 (2)°] indicates the *sp*^2^ and *sp*^3^ hybridized state of N1 and N2 atoms, respectively. The crystal packing is influenced by intermolecular N—H···O, O—H···O and C—H···O interactions (Table 1 & Fig. 2).

### Experimental

The title compound was synthesized by mixing brucine (3.94 g, 0.01 mol) and oxalic acid dihydrate in 50 ml of 50% ethanol/water under reflux for 10 min. The partial room temperature evaporation of the filtered solution gave colorless single crystals in a week.

### Refinement

H atoms were positioned geometrically and refined using riding model with C—H = 0.93 Å and *U*_iso_(H) = 1.2*U*_eq_(C) for aromatic CH, C—H = 0.98 Å and *U*_iso_(H) = 1.2*U*_eq_(C) for CH, C—H = 0.97 Å and *U*_iso_(H) = 1.2*U*_eq_(C) for CH_2_, and C—H = 0.96 Å and *U*_iso_(H) = 1.5*U*_eq_(C) for CH_3_. O- and N-bound H atoms were located in a difference Fourier map and the positions were refined with distance restraints [N2—H2B = 0.88 (1) Å, O5—H5A, O9—H9A, O9—H9B, O10—H10A and O10—H10B = 0.82 (1) Å, and H10C···H10D = 1.40 (2) Å], and with *U*_iso_(H) = 1.5*U*_eq_(O,N).

### Crystal data

**Table:**

C_23_H_27_N_2_O_4_^+^·C_2_HO_4_^−^·2H_2_O	*F*(000) = 1104
*M**_r_* = 520.53	*D*_x_ = 1.434 Mg m^−^^3^
Orthorhombic, *P*2_1_2_1_2_1_	Mo *K*α radiation, λ = 0.71073 Å
Hall symbol: P 2ac 2ab	Cell parameters from 5990 reflections
*a* = 7.6110 (2) Å	θ = 2.0–28.2°
*b* = 10.7375 (3) Å	µ = 0.11 mm^−^^1^
*c* = 29.4990 (7) Å	*T* = 295 K
*V* = 2410.75 (11) Å^3^	Block, colourless
*Z* = 4	0.28 × 0.24 × 0.20 mm

### Data collection

**Table:**

Bruker Kappa APEXII CCD diffractometer	5805 independent reflections
Radiation source: fine-focus sealed tube	5363 reflections with *I* > 2σ(*I*)
Graphite monochromator	*R*_int_ = 0.020
ω and φ scans	θ_max_ = 28.3°, θ_min_ = 2.0°
Absorption correction: multi-scan (*SADABS*; Sheldrick, 1996)	*h* = −4→9
*T*_min_ = 0.970, *T*_max_ = 0.978	*k* = −10→14
13171 measured reflections	*l* = −39→32

### Refinement

**Table:**

Refinement on *F*^2^	Primary atom site location: structure-invariant direct methods
Least-squares matrix: full	Secondary atom site location: difference Fourier map
*R*[*F*^2^ > 2σ(*F*^2^)] = 0.042	Hydrogen site location: inferred from neighbouring sites
*wR*(*F*^2^) = 0.116	H atoms treated by a mixture of independent and constrained refinement
*S* = 1.05	*w* = 1/[σ^2^(*F*_o_^2^) + (0.0708*P*)^2^ + 0.4586*P*] where *P* = (*F*_o_^2^ + 2*F*_c_^2^)/3
5805 reflections	(Δ/σ)_max_ < 0.001
354 parameters	Δρ_max_ = 0.33 e Å^−^^3^
7 restraints	Δρ_min_ = −0.32 e Å^−^^3^

### Special details

**Table:**

Geometry. All e.s.d.'s (except the e.s.d. in the dihedral angle between two l.s. planes) are estimated using the full covariance matrix. The cell e.s.d.'s are taken into account individually in the estimation of e.s.d.'s in distances, angles and torsion angles; correlations between e.s.d.'s in cell parameters are only used when they are defined by crystal symmetry. An approximate (isotropic) treatment of cell e.s.d.'s is used for estimating e.s.d.'s involving l.s. planes.
Refinement. Refinement of *F*^2^ against ALL reflections. The weighted *R*-factor *wR* and goodness of fit *S* are based on *F*^2^, conventional *R*-factors *R* are based on *F*, with *F* set to zero for negative *F*^2^. The threshold expression of *F*^2^ > σ(*F*^2^) is used only for calculating *R*-factors(gt) *etc*. and is not relevant to the choice of reflections for refinement. *R*-factors based on *F*^2^ are statistically about twice as large as those based on *F*, and *R*- factors based on ALL data will be even larger.

### Fractional atomic coordinates and isotropic or equivalent isotropic displacement parameters (Å^2^)

**Table:**

	*x*	*y*	*z*	*U*_iso_*/*U*_eq_	
C1	0.8167 (2)	0.10403 (15)	0.04553 (5)	0.0273 (3)	
C2	0.7938 (2)	0.22682 (15)	0.03194 (5)	0.0300 (3)	
H2	0.7733	0.2890	0.0532	0.036*	
C3	0.8019 (2)	0.25510 (14)	−0.01380 (5)	0.0281 (3)	
C4	0.8270 (2)	0.16002 (15)	−0.04610 (5)	0.0283 (3)	
C5	0.8518 (2)	0.03741 (15)	−0.03262 (5)	0.0277 (3)	
H5	0.8702	−0.0256	−0.0537	0.033*	
C6	0.8480 (2)	0.01221 (14)	0.01373 (5)	0.0262 (3)	
C7	0.8725 (2)	−0.08541 (14)	0.08561 (5)	0.0240 (3)	
H7	0.7940	−0.1469	0.0995	0.029*	
C8	0.7954 (2)	0.04770 (14)	0.09221 (5)	0.0255 (3)	
C9	0.9615 (3)	−0.20278 (15)	0.01824 (6)	0.0331 (4)	
C10	1.0233 (3)	−0.29667 (16)	0.05273 (6)	0.0407 (4)	
H10A	1.1123	−0.3482	0.0385	0.049*	
H10B	0.9249	−0.3505	0.0600	0.049*	
C11	1.0995 (2)	−0.24681 (16)	0.09759 (6)	0.0330 (4)	
H11	1.2270	−0.2597	0.0978	0.040*	
C12	1.0603 (2)	−0.10686 (15)	0.10212 (5)	0.0263 (3)	
H12	1.1366	−0.0652	0.0801	0.032*	
C13	1.0974 (2)	−0.04399 (16)	0.14785 (6)	0.0287 (3)	
H13	1.2200	−0.0594	0.1563	0.034*	
C14	1.0718 (2)	0.09617 (16)	0.14006 (6)	0.0331 (4)	
H14A	1.1111	0.1422	0.1665	0.040*	
H14B	1.1406	0.1230	0.1142	0.040*	
C15	0.8791 (2)	0.12124 (15)	0.13160 (5)	0.0288 (3)	
H2A	0.8636	0.2104	0.1258	0.035*	
C16	0.6084 (2)	0.0154 (2)	0.15679 (6)	0.0371 (4)	
H16A	0.6202	−0.0731	0.1625	0.045*	
H16B	0.5032	0.0455	0.1718	0.045*	
C17	0.6010 (2)	0.04159 (18)	0.10656 (6)	0.0346 (4)	
H17A	0.5420	0.1199	0.1006	0.042*	
H17B	0.5400	−0.0245	0.0906	0.042*	
C18	1.0805 (4)	−0.30313 (19)	0.17721 (7)	0.0494 (5)	
H18A	1.2029	−0.2779	0.1768	0.059*	
H18B	1.0718	−0.3800	0.1943	0.059*	
C19	0.9727 (3)	−0.20421 (18)	0.19990 (6)	0.0387 (4)	
H19	0.9001	−0.2256	0.2240	0.046*	
C20	0.9792 (2)	−0.08679 (16)	0.18616 (5)	0.0296 (3)	
C21	0.8705 (3)	0.01255 (17)	0.20833 (6)	0.0329 (4)	
H21A	0.9465	0.0685	0.2251	0.040*	
H21B	0.7893	−0.0254	0.2296	0.040*	
C22	0.8296 (3)	0.47074 (16)	−0.00029 (7)	0.0383 (4)	
H22A	0.7379	0.4784	0.0219	0.057*	
H22B	0.8411	0.5477	−0.0166	0.057*	
H22C	0.9385	0.4518	0.0146	0.057*	
C23	0.8254 (4)	0.1045 (2)	−0.12426 (7)	0.0518 (6)	
H23A	0.9309	0.0558	−0.1224	0.078*	
H23B	0.8183	0.1428	−0.1536	0.078*	
H23C	0.7253	0.0515	−0.1198	0.078*	
C24	0.6076 (2)	0.36823 (15)	0.23109 (6)	0.0334 (4)	
C25	0.6212 (3)	0.51090 (15)	0.23589 (6)	0.0337 (4)	
N1	0.8757 (2)	−0.10346 (12)	0.03567 (4)	0.0277 (3)	
N2	0.7689 (2)	0.08528 (14)	0.17329 (5)	0.0309 (3)	
H2B	0.728 (3)	0.1521 (16)	0.1869 (8)	0.046*	
O1	0.8278 (2)	0.19783 (12)	−0.09044 (4)	0.0388 (3)	
O2	0.7871 (2)	0.37348 (11)	−0.03112 (4)	0.0376 (3)	
O3	0.9853 (3)	−0.21823 (13)	−0.02266 (5)	0.0509 (4)	
O4	1.0205 (2)	−0.32384 (12)	0.13148 (5)	0.0432 (3)	
O5	0.4765 (2)	0.57002 (13)	0.22834 (7)	0.0586 (5)	
H5A	0.470 (5)	0.6456 (12)	0.2345 (12)	0.088*	
O6	0.7510 (3)	0.56036 (16)	0.24876 (10)	0.0912 (9)	
O7	0.5180 (3)	0.31491 (13)	0.25994 (6)	0.0654 (6)	
O8	0.6928 (2)	0.32090 (14)	0.19997 (5)	0.0524 (4)	
O9	0.5505 (4)	0.7048 (3)	0.11705 (8)	0.0835 (6)	
H9A	0.565 (6)	0.709 (5)	0.0885 (4)	0.125*	
H9B	0.609 (6)	0.647 (3)	0.1290 (16)	0.125*	
O10	0.7913 (4)	0.4655 (3)	0.12287 (7)	0.0885 (8)	
H10C	0.8980 (17)	0.462 (5)	0.1196 (15)	0.133*	
H10D	0.758 (5)	0.429 (4)	0.1460 (10)	0.133*	

### Atomic displacement parameters (Å^2^)

**Table:**

	*U*^11^	*U*^22^	*U*^33^	*U*^12^	*U*^13^	*U*^23^
C1	0.0343 (8)	0.0252 (7)	0.0223 (7)	0.0027 (6)	−0.0002 (6)	0.0020 (6)
C2	0.0409 (9)	0.0237 (7)	0.0255 (7)	0.0035 (6)	−0.0005 (7)	−0.0003 (6)
C3	0.0350 (8)	0.0222 (7)	0.0271 (7)	0.0006 (6)	−0.0029 (6)	0.0033 (6)
C4	0.0333 (8)	0.0287 (8)	0.0228 (7)	−0.0018 (6)	−0.0025 (6)	0.0023 (6)
C5	0.0357 (8)	0.0249 (7)	0.0226 (7)	0.0009 (6)	−0.0023 (6)	−0.0018 (6)
C6	0.0325 (8)	0.0206 (7)	0.0254 (7)	0.0007 (6)	−0.0011 (6)	0.0011 (5)
C7	0.0295 (7)	0.0202 (6)	0.0224 (6)	−0.0010 (6)	0.0007 (6)	0.0010 (5)
C8	0.0331 (8)	0.0204 (7)	0.0229 (7)	0.0035 (6)	0.0010 (6)	0.0023 (5)
C9	0.0472 (10)	0.0236 (7)	0.0285 (8)	0.0034 (7)	−0.0008 (7)	−0.0012 (6)
C10	0.0663 (12)	0.0236 (7)	0.0323 (9)	0.0115 (8)	−0.0008 (9)	−0.0030 (6)
C11	0.0396 (9)	0.0279 (8)	0.0314 (8)	0.0098 (7)	0.0000 (7)	0.0013 (6)
C12	0.0283 (7)	0.0251 (7)	0.0255 (7)	−0.0002 (6)	0.0022 (6)	0.0004 (6)
C13	0.0259 (7)	0.0318 (8)	0.0283 (8)	−0.0029 (6)	−0.0018 (6)	−0.0003 (6)
C14	0.0399 (9)	0.0283 (8)	0.0310 (8)	−0.0122 (7)	0.0024 (7)	−0.0029 (6)
C15	0.0433 (9)	0.0205 (7)	0.0225 (7)	−0.0008 (7)	0.0041 (7)	0.0005 (5)
C16	0.0312 (8)	0.0506 (11)	0.0294 (8)	−0.0005 (8)	0.0047 (7)	0.0042 (8)
C17	0.0328 (8)	0.0428 (9)	0.0283 (8)	0.0081 (7)	0.0010 (7)	0.0028 (7)
C18	0.0812 (16)	0.0349 (9)	0.0321 (9)	0.0151 (10)	−0.0075 (10)	0.0069 (8)
C19	0.0514 (11)	0.0378 (9)	0.0270 (8)	−0.0005 (8)	−0.0003 (8)	0.0044 (7)
C20	0.0323 (8)	0.0326 (8)	0.0239 (7)	−0.0007 (7)	−0.0021 (6)	0.0003 (6)
C21	0.0397 (9)	0.0360 (9)	0.0231 (7)	0.0019 (7)	0.0019 (7)	0.0015 (6)
C22	0.0549 (11)	0.0242 (8)	0.0357 (9)	−0.0015 (8)	−0.0059 (8)	−0.0002 (7)
C23	0.0891 (17)	0.0400 (10)	0.0264 (8)	0.0080 (11)	−0.0042 (10)	−0.0017 (8)
C24	0.0441 (9)	0.0212 (7)	0.0350 (8)	0.0023 (7)	0.0045 (7)	0.0003 (6)
C25	0.0417 (9)	0.0229 (7)	0.0366 (9)	−0.0005 (7)	0.0056 (8)	0.0019 (6)
N1	0.0382 (7)	0.0221 (6)	0.0229 (6)	0.0002 (6)	−0.0022 (5)	0.0005 (5)
N2	0.0378 (7)	0.0315 (7)	0.0233 (6)	0.0054 (6)	0.0036 (6)	−0.0006 (5)
O1	0.0630 (9)	0.0298 (6)	0.0236 (6)	0.0004 (6)	−0.0020 (6)	0.0036 (5)
O2	0.0614 (8)	0.0229 (5)	0.0285 (6)	−0.0004 (6)	−0.0094 (6)	0.0038 (5)
O3	0.0907 (12)	0.0356 (7)	0.0264 (6)	0.0144 (8)	0.0030 (7)	−0.0031 (5)
O4	0.0689 (10)	0.0273 (6)	0.0333 (7)	0.0015 (6)	−0.0040 (7)	0.0054 (5)
O5	0.0460 (8)	0.0263 (6)	0.1035 (14)	0.0053 (6)	0.0038 (9)	0.0004 (8)
O6	0.0656 (12)	0.0400 (9)	0.168 (3)	−0.0093 (9)	−0.0456 (15)	0.0125 (12)
O7	0.1001 (14)	0.0238 (6)	0.0724 (12)	−0.0072 (8)	0.0471 (11)	−0.0007 (7)
O8	0.0770 (11)	0.0329 (7)	0.0473 (8)	0.0084 (7)	0.0221 (8)	−0.0039 (6)
O9	0.0839 (15)	0.0941 (17)	0.0725 (14)	−0.0141 (14)	−0.0099 (12)	0.0102 (13)
O10	0.1118 (19)	0.0983 (17)	0.0553 (11)	−0.0444 (16)	0.0036 (12)	0.0131 (11)

### Geometric parameters (Å, º)

**Table:**

C1—C6	1.382 (2)	C15—H2A	0.9800
C1—C2	1.389 (2)	C16—C17	1.509 (2)
C1—C8	1.513 (2)	C16—N2	1.514 (2)
C2—C3	1.384 (2)	C16—H16A	0.9700
C2—H2	0.9300	C16—H16B	0.9700
C3—O2	1.3745 (19)	C17—H17A	0.9700
C3—C4	1.410 (2)	C17—H17B	0.9700
C4—O1	1.3694 (19)	C18—O4	1.441 (2)
C4—C5	1.388 (2)	C18—C19	1.499 (3)
C5—C6	1.394 (2)	C18—H18A	0.9700
C5—H5	0.9300	C18—H18B	0.9700
C6—N1	1.4162 (19)	C19—C20	1.325 (2)
C7—N1	1.4861 (19)	C19—H19	0.9300
C7—C12	1.528 (2)	C20—C21	1.500 (2)
C7—C8	1.557 (2)	C21—N2	1.509 (2)
C7—H7	0.9800	C21—H21A	0.9700
C8—C17	1.540 (2)	C21—H21B	0.9700
C8—C15	1.543 (2)	C22—O2	1.422 (2)
C9—O3	1.231 (2)	C22—H22A	0.9600
C9—N1	1.352 (2)	C22—H22B	0.9600
C9—C10	1.508 (2)	C22—H22C	0.9600
C10—C11	1.541 (3)	C23—O1	1.414 (2)
C10—H10A	0.9700	C23—H23A	0.9600
C10—H10B	0.9700	C23—H23B	0.9600
C11—O4	1.430 (2)	C23—H23C	0.9600
C11—C12	1.538 (2)	C24—O7	1.232 (2)
C11—H11	0.9800	C24—O8	1.234 (2)
C12—C13	1.535 (2)	C24—C25	1.542 (2)
C12—H12	0.9800	C25—O6	1.184 (3)
C13—C20	1.515 (2)	C25—O5	1.291 (3)
C13—C14	1.535 (2)	N2—H2B	0.880 (10)
C13—H13	0.9800	O5—H5A	0.833 (10)
C14—C15	1.512 (3)	O9—H9A	0.850 (10)
C14—H14A	0.9700	O9—H9B	0.836 (10)
C14—H14B	0.9700	O10—H10C	0.818 (10)
C15—N2	1.537 (2)	O10—H10D	0.824 (10)
			
C6—C1—C2	120.21 (15)	C14—C15—H2A	108.6
C6—C1—C8	110.56 (13)	N2—C15—H2A	108.6
C2—C1—C8	128.96 (14)	C8—C15—H2A	108.6
C3—C2—C1	118.94 (15)	C17—C16—N2	104.69 (14)
C3—C2—H2	120.5	C17—C16—H16A	110.8
C1—C2—H2	120.5	N2—C16—H16A	110.8
O2—C3—C2	124.15 (15)	C17—C16—H16B	110.8
O2—C3—C4	115.44 (14)	N2—C16—H16B	110.8
C2—C3—C4	120.40 (14)	H16A—C16—H16B	108.9
O1—C4—C5	123.65 (14)	C16—C17—C8	104.00 (13)
O1—C4—C3	115.56 (14)	C16—C17—H17A	111.0
C5—C4—C3	120.78 (14)	C8—C17—H17A	111.0
C4—C5—C6	117.53 (14)	C16—C17—H17B	111.0
C4—C5—H5	121.2	C8—C17—H17B	111.0
C6—C5—H5	121.2	H17A—C17—H17B	109.0
C1—C6—C5	122.07 (14)	O4—C18—C19	110.72 (17)
C1—C6—N1	109.96 (14)	O4—C18—H18A	109.5
C5—C6—N1	127.97 (14)	C19—C18—H18A	109.5
N1—C7—C12	106.32 (13)	O4—C18—H18B	109.5
N1—C7—C8	104.47 (12)	C19—C18—H18B	109.5
C12—C7—C8	116.82 (13)	H18A—C18—H18B	108.1
N1—C7—H7	109.6	C20—C19—C18	121.13 (18)
C12—C7—H7	109.6	C20—C19—H19	119.4
C8—C7—H7	109.6	C18—C19—H19	119.4
C1—C8—C17	111.72 (13)	C19—C20—C21	121.50 (16)
C1—C8—C15	115.90 (13)	C19—C20—C13	122.60 (16)
C17—C8—C15	102.20 (13)	C21—C20—C13	115.90 (14)
C1—C8—C7	102.29 (12)	C20—C21—N2	110.60 (13)
C17—C8—C7	110.93 (13)	C20—C21—H21A	109.5
C15—C8—C7	114.10 (13)	N2—C21—H21A	109.5
O3—C9—N1	123.36 (16)	C20—C21—H21B	109.5
O3—C9—C10	121.68 (16)	N2—C21—H21B	109.5
N1—C9—C10	114.94 (15)	H21A—C21—H21B	108.1
C9—C10—C11	117.69 (14)	O2—C22—H22A	109.5
C9—C10—H10A	107.9	O2—C22—H22B	109.5
C11—C10—H10A	107.9	H22A—C22—H22B	109.5
C9—C10—H10B	107.9	O2—C22—H22C	109.5
C11—C10—H10B	107.9	H22A—C22—H22C	109.5
H10A—C10—H10B	107.2	H22B—C22—H22C	109.5
O4—C11—C12	115.02 (14)	O1—C23—H23A	109.5
O4—C11—C10	103.96 (15)	O1—C23—H23B	109.5
C12—C11—C10	109.95 (14)	H23A—C23—H23B	109.5
O4—C11—H11	109.2	O1—C23—H23C	109.5
C12—C11—H11	109.2	H23A—C23—H23C	109.5
C10—C11—H11	109.2	H23B—C23—H23C	109.5
C7—C12—C13	112.69 (13)	O7—C24—O8	127.87 (17)
C7—C12—C11	107.52 (13)	O7—C24—C25	115.82 (16)
C13—C12—C11	118.08 (14)	O8—C24—C25	116.25 (16)
C7—C12—H12	105.9	O6—C25—O5	123.14 (17)
C13—C12—H12	105.9	O6—C25—C24	122.07 (18)
C11—C12—H12	105.9	O5—C25—C24	114.56 (17)
C20—C13—C12	114.40 (13)	C9—N1—C6	126.16 (14)
C20—C13—C14	109.49 (14)	C9—N1—C7	119.19 (13)
C12—C13—C14	106.05 (13)	C6—N1—C7	109.64 (12)
C20—C13—H13	108.9	C21—N2—C16	112.17 (14)
C12—C13—H13	108.9	C21—N2—C15	113.49 (14)
C14—C13—H13	108.9	C16—N2—C15	107.91 (13)
C15—C14—C13	108.81 (13)	C21—N2—H2B	106.9 (16)
C15—C14—H14A	109.9	C16—N2—H2B	105.2 (17)
C13—C14—H14A	109.9	C15—N2—H2B	110.8 (17)
C15—C14—H14B	109.9	C4—O1—C23	117.63 (14)
C13—C14—H14B	109.9	C3—O2—C22	115.02 (13)
H14A—C14—H14B	108.3	C11—O4—C18	115.61 (16)
C14—C15—N2	110.62 (13)	C25—O5—H5A	120 (3)
C14—C15—C8	115.71 (14)	H9A—O9—H9B	113 (5)
N2—C15—C8	104.41 (13)	H10C—O10—H10D	112 (3)
			
C6—C1—C2—C3	0.4 (3)	C17—C8—C15—C14	153.36 (14)
C8—C1—C2—C3	−173.02 (16)	C7—C8—C15—C14	33.55 (18)
C1—C2—C3—O2	−177.73 (17)	C1—C8—C15—N2	153.27 (13)
C1—C2—C3—C4	2.1 (3)	C17—C8—C15—N2	31.53 (15)
O2—C3—C4—O1	−2.0 (2)	C7—C8—C15—N2	−88.28 (15)
C2—C3—C4—O1	178.14 (16)	N2—C16—C17—C8	34.94 (18)
O2—C3—C4—C5	177.02 (16)	C1—C8—C17—C16	−165.99 (14)
C2—C3—C4—C5	−2.9 (3)	C15—C8—C17—C16	−41.43 (17)
O1—C4—C5—C6	179.91 (16)	C7—C8—C17—C16	80.57 (17)
C3—C4—C5—C6	1.0 (2)	O4—C18—C19—C20	−67.2 (3)
C2—C1—C6—C5	−2.3 (3)	C18—C19—C20—C21	179.35 (18)
C8—C1—C6—C5	172.24 (15)	C18—C19—C20—C13	−1.5 (3)
C2—C1—C6—N1	177.02 (16)	C12—C13—C20—C19	60.4 (2)
C8—C1—C6—N1	−8.48 (19)	C14—C13—C20—C19	179.25 (17)
C4—C5—C6—C1	1.6 (2)	C12—C13—C20—C21	−120.38 (16)
C4—C5—C6—N1	−177.60 (16)	C14—C13—C20—C21	−1.5 (2)
C6—C1—C8—C17	−102.75 (17)	C19—C20—C21—N2	−128.70 (18)
C2—C1—C8—C17	71.1 (2)	C13—C20—C21—N2	52.1 (2)
C6—C1—C8—C15	140.73 (15)	O7—C24—C25—O6	106.6 (3)
C2—C1—C8—C15	−45.4 (2)	O8—C24—C25—O6	−70.8 (3)
C6—C1—C8—C7	15.96 (18)	O7—C24—C25—O5	−68.1 (3)
C2—C1—C8—C7	−170.16 (17)	O8—C24—C25—O5	114.5 (2)
N1—C7—C8—C1	−16.91 (15)	O3—C9—N1—C6	−20.4 (3)
C12—C7—C8—C1	100.19 (15)	C10—C9—N1—C6	161.06 (17)
N1—C7—C8—C17	102.36 (14)	O3—C9—N1—C7	−172.66 (18)
C12—C7—C8—C17	−140.54 (14)	C10—C9—N1—C7	8.8 (2)
N1—C7—C8—C15	−142.87 (13)	C1—C6—N1—C9	−157.88 (17)
C12—C7—C8—C15	−25.77 (19)	C5—C6—N1—C9	21.4 (3)
O3—C9—C10—C11	141.4 (2)	C1—C6—N1—C7	−3.42 (19)
N1—C9—C10—C11	−40.0 (3)	C5—C6—N1—C7	175.81 (16)
C9—C10—C11—O4	135.72 (18)	C12—C7—N1—C9	45.57 (19)
C9—C10—C11—C12	12.1 (2)	C8—C7—N1—C9	169.70 (15)
N1—C7—C12—C13	157.39 (12)	C12—C7—N1—C6	−110.93 (14)
C8—C7—C12—C13	41.30 (18)	C8—C7—N1—C6	13.20 (17)
N1—C7—C12—C11	−70.77 (15)	C20—C21—N2—C16	78.03 (17)
C8—C7—C12—C11	173.15 (13)	C20—C21—N2—C15	−44.60 (19)
O4—C11—C12—C7	−75.91 (18)	C17—C16—N2—C21	−140.63 (14)
C10—C11—C12—C7	41.01 (19)	C17—C16—N2—C15	−14.89 (18)
O4—C11—C12—C13	52.9 (2)	C14—C15—N2—C21	−10.95 (19)
C10—C11—C12—C13	169.84 (15)	C8—C15—N2—C21	114.17 (15)
C7—C12—C13—C20	59.13 (18)	C14—C15—N2—C16	−135.90 (15)
C11—C12—C13—C20	−67.24 (19)	C8—C15—N2—C16	−10.78 (17)
C7—C12—C13—C14	−61.65 (17)	C5—C4—O1—C23	10.6 (3)
C11—C12—C13—C14	171.98 (14)	C3—C4—O1—C23	−170.48 (19)
C20—C13—C14—C15	−55.45 (18)	C2—C3—O2—C22	23.2 (3)
C12—C13—C14—C15	68.45 (17)	C4—C3—O2—C22	−156.72 (17)
C13—C14—C15—N2	62.41 (18)	C12—C11—O4—C18	−64.4 (2)
C13—C14—C15—C8	−56.03 (18)	C10—C11—O4—C18	175.37 (16)
C1—C8—C15—C14	−84.91 (17)	C19—C18—O4—C11	88.6 (2)

### Hydrogen-bond geometry (Å, º)

**Table:**

*D*—H···*A*	*D*—H	H···*A*	*D*···*A*	*D*—H···*A*
N2—H2*B*···O8	0.88 (1)	1.87 (1)	2.712 (2)	159 (2)
O5—H5*A*···O7^i^	0.83 (1)	1.83 (1)	2.652 (2)	170 (4)
O9—H9*A*···O3^ii^	0.85 (1)	2.04 (2)	2.832 (3)	155 (5)
O9—H9*B*···O10	0.84 (1)	2.40 (3)	3.161 (4)	151 (5)
O10—H10*D*···O8	0.82 (1)	2.04 (1)	2.854 (3)	173 (5)
C7—H7···O9^iii^	0.98	2.50	3.456 (3)	166
C11—H11···O9^iv^	0.98	2.56	3.519 (3)	168
C17—H17*A*···O1^ii^	0.97	2.56	3.518 (2)	168
C21—H21*A*···O6^v^	0.97	2.43	3.188 (3)	135
C21—H21*B*···O5^vi^	0.97	2.59	3.293 (3)	130
C14—H14*B*···O1^vii^	0.97	2.49	3.291 (2)	139

Symmetry codes: (i) −*x*+1, *y*+1/2, −*z*+1/2; (ii) *x*−1/2, −*y*+1/2, −*z*; (iii) *x*, *y*−1, *z*; (iv) *x*+1, *y*−1, *z*; (v) −*x*+2, *y*−1/2, −*z*+1/2; (vi) −*x*+1, *y*−1/2, −*z*+1/2; (vii) *x*+1/2, −*y*+1/2, −*z*.
